# Severe Guillain–Barré syndrome associated with chronic hepatitis B: A case report and literature review

**DOI:** 10.1097/MD.0000000000027989

**Published:** 2021-12-03

**Authors:** Jiajun Wei, Shenhan Duan

**Affiliations:** Department of Neurology, Renmin Hospital of Wuhan University, Wuhan, Hubei, China.

**Keywords:** acute exacerbation, chronic hepatitis B, extrahepatic manifestation, Guillain–Barré syndrome

## Abstract

**Rationale:**

Guillain–Barré syndrome (GBS) is a postinfectious autoimmune peripheral neuropathy characterized by acute paralysis of the limbs. Clinically, extrahepatic manifestations of neurologic involvement in chronic hepatitis B (CHB) are uncommon. Little attention has been paid to the relationship between GBS and CHB viral infection.

**Patient concerns:**

We presented a severe case of a 34-year-old man with general fatigue, anorexia, jaundice, numbness, and even muscle atrophy in the limbs, and respiratory failure during an acute exacerbation of CHB.

**Diagnoses:**

Serological liver enzymes test confirmed an acute exacerbation of CHB. Nerve conduction studies revealed the features of acute motor and sensory axonal neuropathy combined with acute inflammatory demyelinating polyneuropathy, and cerebrospinal fluid analysis showed albuminocytologic dissociation. Clinical manifestations and the test results were consistent with a diagnosis of severe CHB-related GBS.

**Interventions:**

He was treated with mechanical ventilation, 2 courses of intravenous immunoglobulin, antichronic hepatitis B drugs therapy supplemented by hepatoprotection, acupuncture and rehabilitation.

**Outcomes:**

After 29 days of hospitalization, his neurological condition improved. At a 6-month follow-up visit, he was able to walk with the support of another person.

**Lessons:**

The acute exacerbation of CHB may be a potential predisposing factor for the onset of GBS. This case is a reminder to clinicians that during the acute exacerbation of CHB, patients with neurological symptoms in the limbs should be considered for potential CHB-related GBS.

## 1 Introduction

Chronic hepatitis B (CHB) is a chronic inflammatory disease of the liver caused by persistent hepatitis B virus (HBV) infection. The World Health Organization estimated that there were about 257 million people with chronic HBV infection in 2015.^[1]^ Of these, 68% were distributed in Africa and the Western Pacific regions. Approximately 650,000 people worldwide die each year from complications of CHB such as cirrhosis and hepatocellular carcinoma.^[2]^ In China, there are currently about 70 million people infected with HBV, including nearly 20 million to 30 million cases with CHB,^[1,3]^ which places a heavy burden on both patients’ families and society. In addition to the common manifestations of the affected liver such as icteric hepatitis, ascites, cirrhosis and liver cancer, rare extrahepatic manifestations associated with HBV infection have also been reported including arthritis, polyarteritis nodosa, glomerulonephritis, papular dermatitis,^[4]^ and easily overlooked manifestations of neurological damage such as encephalitis, myelitis, pyramidal and extrapyramidal dysfunction, mental illness, epileptic seizures, neuritis and dermatomyositis.^[5]^ However, only a few cases of CHB-related Guillain–Barré syndrome (GBS) have been reported.^[4,6–8]^ GBS is a rare postinfectious autoimmune condition with acute tetraplegic polyradiculoneuropathy and an incidence of approximately 1.1 per 100,000 individuals.^[9]^ It may occur in both children and adults, and when severe, can lead to death from respiratory failure. Some common infectious agents that cause GBS include *Campylobacter jejuni*, *Mycoplasma pneumoniae*, cytomegalovirus, and Epstein–Barr virus.^[10]^ However, chronic HBV infection triggering the occurrence of GBS is rare. Clinicians know little about the clinical course of GBS due to CHB, and have difficulty fully understanding the association between them. In October 2019, we encountered 1 patient with severe GBS superimposed on acute exacerbation of CHB. To our knowledge, this is the first case of GBS related to CHB reported in English and occurring in Chinese mainland. In addition, we reviewed the English literature on CHB-related GBS, aiming to improve clinicians’ understanding and early recognition of the disease.

## 2 Case presentation

A 34-year-old male patient complained of general fatigue, anorexia, nausea and jaundice with slight weakness and numbness in the limbs for 2 weeks. He had a 6-year history of chronic schizophrenia with long-term olanzapine or aripiprazole maintenance therapy. He was diagnosed with CHB by infectious disease specialists due to the development of symptoms of malaise and jaundice 2 years prior. Half a year later he was treated with entecavir (ETV) for 11 months, and then self-discontinued therapy owing to symptom improvement. He had no history of blood transfusion, drug abuse, high-risk sexual behavior, or travel abroad. Initially, he was referred to the infectious disease clinic at a local hospital where serological tests showed positive hepatitis B surface antigen (HBsAg), an alanine aminotransferase (ALT) level of 162 IU/L (9–50 IU/L), and an aspartate transaminase (AST) level of 121 IU/L (9–50 IU/L). The patient still had a diagnosis of CHB and was treated with hepatoprotective therapy in that outpatient clinic. However, after 5 days of treatment, he had no obvious improvement in symptoms of fatigue, jaundice, difficulty climbing stairs, numbness in both hands, and weak grip strength, and subsequently he was transferred to the Department of Infectious Disease at our hospital for further treatment.

On the day of admission, his general medical examination revealed uneventful vital signs, yellowish skin, slight percussive pain in the liver area, no visible liver palms or spider nevi, and no obvious abnormalities in heart and lung auscultation. Laboratory examination showed a normal blood routine, serum AST/ALT levels of 231/204 IU/L, 32.7 g/L albumin (40–55 g/L), 24.8 g/L globulin (20–40 g/L), 5.0 mg/dL total bilirubin (0–1.4 mg/dL), 3.8 mg/dL direct bilirubin (0–0.5 mg/dL), 8.3 ng/mL alpha-fetoprotein (0–8.1 ng/mL), and 22.7 μmol/L blood ammonia (11–32 μmol/L). A serological hepatitis B marker test showed positive HBsAg, negative hepatitis B e-antigen (HBeAg), positive HBe-antibody (HBeAb), positive hepatitis B core antibody (HBcAb), 1:502 IgM anti-HBc antibody quantification, 1.1 × 10^5^ IU/mL HBV-DNA blood viral load, and negative for all other serological markers of hepatitis (including hepatitis A, C, D and E). The Fibro Test–Acti Test Panel revealed a mild to moderate fibrosis stage (F1–F2) and mild to moderate activity grade (A1–A2). He was seronegative for microbial antibodies to *Treponema pallidum*, human immunodeficiency virus, *C jejuni*, rubella virus, Epstein–Barr virus, cytomegalovirus and herpes simplex virus, and ganglioside (GD1a, GQ1b, GM1, and GM2) antibodies.

**Figure 1:**
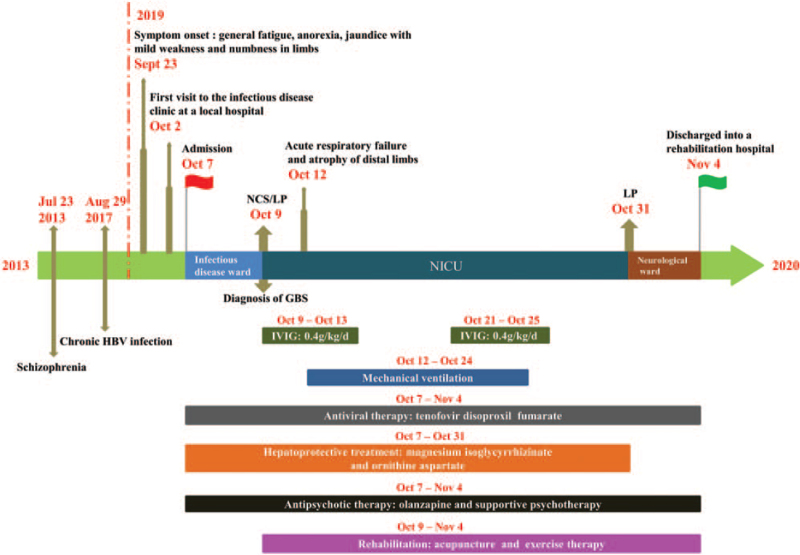
Medical history and theragnostic course of a CHB-related GBS patient. CHB = chronic hepatitis B, GBS = Guillain–Barré syndrome, HBV = hepatitis B virus, IVIG = intravenous immunoglobulin, LP = lumbar puncture, NCS = nerve conduction studies, NICU = neurology intensive care unit.

However, by day 3 of admission, the limb muscle weakness and numbness were increasing and he was unable to walk independently, with no breathing and swallowing difficulties. The patient underwent immediate consultation with a neurologist, and the physical examination revealed the following: proximal grade 3/5 and distal grade 2/5 muscle strength in his limbs with low muscle tone, and diminished bilateral brachial biceps reflex, triceps reflex, knee reflex, and Achilles tendon reflex. The temperature sensation and pain sensation predominantly in his distal limbs were decreased, and his Babinski sign in both lower limbs was negative. No cranial nerve palsy, aphasia, or disturbance of consciousness was observed. The Medical Research Council sum score (MRC-sumscore), assessing the strength of 6 pairs of individual muscles in the 4 limbs according to the MRC scale and ranging from 0 (paralysis) to 60 (normal strength),^[11]^ was 28/60. The neurological physical examination supported the working diagnosis of GBS, and he was transferred to the Neurology Intensive Care Unit for continued treatment. On the same day, a recheck of serum liver function showed that the ALT/AST levels (723/533 IU/L) had risen to more than 10 times the upper limit of normal, suggesting acute exacerbation of CHB. Nerve conduction studies (NCS) showed extensive demyelination and axonal damage to the distal and proximal sensorimotor fibers and nerve roots of the peripheral nerves in his limbs, revealing evidence of acute motor sensory axonal neuropathy (AMSAN) with concomitant acute inflammatory demyelinating polyneuropathy (AIDP) compatible with a severe type of GBS (Table 1). The cerebrospinal fluid (CSF) examination showed a white blood cell count of 2 × 10^6^/L, a red blood cell count of 0 × 10^9^/L, 3.94 mmol/L glucose, and 0.72 g/L protein, indicating the existence of albuminocytologic dissociation. No noticeable abnormalities on magnetic resonance imaging of the head and neck were found. Abdominal B-ultrasonography showed diffuse liver parenchymal lesions, mild spleen enlargement, and no ascites. Figure 1 summarizes the clinical course of the patient.

**Table 1:** Nerve conduction studies in the patient with CHB-related GBS.

		Latency (ms)		Amplitude^∗^		Conduction velocity (m/s)		F-mean latency (ms)	
Nerve	Stimulation site	RT	LT	RT	LT	RT	LT	RT	LT
Median (s)	Wrist – Digit II	NR	NR	NR	NR	NR	NR		
Radial (s)	EPL tendon – Wrist	NR	NR	NR	NR	NR	NR		
Ulnar (s)	Wrist – Digit V	1.92	2.01	4.20^†^	3.20^†^	62.5	62.2		
Superficial peroneal (s)	Lower leg – Ankle	2.07	2.03	7.60^†^	9.60^†^	43.5	49.3		
Sural (s)	Mid lower leg – Lat Malleolus	2.01	1.90	8.30^†^	12.0	49.8	42.1		
Median (m)	Wrist – APB	4.37^‡^	4.29^‡^	0.46^†^	0.25^†^			NR	NR
	Elbow – Wrist	9.72	8.99	0.12^†^	0.10^†^	51.4	56.4		
Ulnar (m)	Wrist – ADM	2.38	2.63	4.80^†^	4.20^†^			31.40^‡^	31.30^‡^
	Bl. elbow – Wrist	7.04	7.15	3.50^†^	3.20^†^	54.7	53.4		
	Ab. elbow – Bl elbow	8.79	9.06	2.20^†^	2.70^†^	54.3	52.4		
Peroneal (m)	Ankle – EDB	4.84	5.08^‡^	0.16^†^	0.96^†^				
	Bl. Fib. head – Ankle	14.0	12.40	0.13^†^	0.59^†^	40.4	47.8		
Tibial (m)	Ankle – Abd hal	4.33	3.63	6.90	6.60			63.20^‡^	60.20^‡^

**Figure 2:**
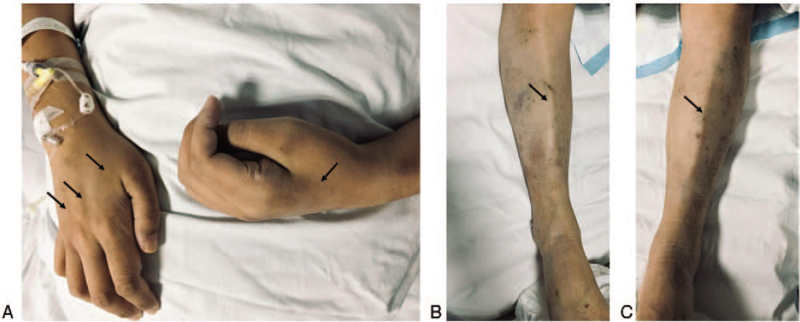
Muscle atrophy of the limbs. A: The obvious atrophy of dorsal interosseous muscles (arrow) in both hands. B and C: The obvious exposure of tibial anterior margin (arrow) in both lower legs.

**Figure 3:**
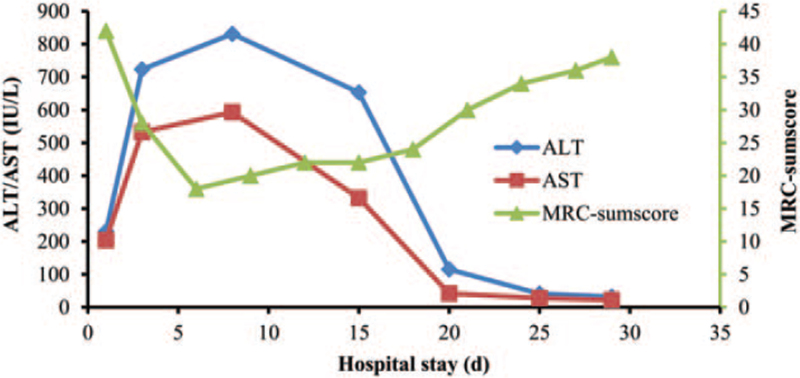
Liver function and MRC-sumscore during hospitalisation. ALT = alanine aminotransferase, AST = aspartate transaminase, MRC = Medical Research Council.

The patient's clinical history, physical examination, serum biochemistry, CSF analysis, and electromyography results supported the diagnosis of CHB-related GBS. He was then given a nucleotide analogue (NA), tenofovir disoproxil fumarate (TDF), 300 mg/d orally to inhibit viral replication, intravenous drip of 0.1 g/d magnesium isoglycyrrhizinate for liver protection, 0.4 g/kg/d intravenous immunoglobulin (IVIG) for 5 consecutive days, intramuscular injection of vitamin B1 and B12, and other treatments including 5 mg/d oral olanzapine and psychological supportive therapy for schizophrenia. Unfortunately, the neurological symptoms of this patient continued to deteriorate. By the 6^th^ day of admission, his muscle strength fell to grade 2+/5 in the proximal limbs and grade 2−/5 in the distal limbs; muscle atrophy predominantly in the distal limbs was evident (Fig. 2). He also developed dyspnea, tachypnea, delirium, and a rapid drop in pulse oxygen saturation to 82%. Due to the acute respiratory failure, he was immediately administered a tracheotomy and ventilation as supportive respiratory therapy (Hughes GBS disability grade 5/6, MRC-sumscore 18/60). At the same time, the serum liver enzyme level continued to rise, and by day 8 of admission, the ALT/AST levels rose to 831/593 IU/L and blood ammonia increased to 82.0 μmol/L. Intravenous drip of ornithine aspartate (10 g/d) was added immediately to strengthen liver protection and lower the blood ammonia level. Because the patient's condition improved slowly, a second course of IVIG was administered 1 week after the end of the first course treatment. In addition, acupuncture and limb function rehabilitation were performed as soon as vital signs became relatively stable. Afterward, neurological symptoms ceased deteriorating, limb muscle strength and respiratory function improved slowly, and serum liver enzyme level gradually decreased. By day 25 of admission, his serum ALT/AST (41/28 IU/L) and CSF protein levels resumed to normal, and the limb muscle strength MRC-sumscore returned to 34/60. He was then transferred to the general ward. By day 29 of admission and prior to discharge, he was in a stable mental state, generalized jaundice had resolved, serum liver function was normal, his spontaneous breathing was smooth, and he had partial recovery of muscle strength in the limbs (proximal: grade 3+/5, distal: grade 3/5). He could stand with support, yet left the muscle atrophy in his distal limbs (Hughes GBS disability grade 4/6, MRC-sumscore 38/60). Figure 3 summarizes the serum liver enzyme level and muscle strength MRC-sumscore of the limbs during hospitalization. At the 6-month follow-up after discharge, the patient was still adhering to acupuncture and limb rehabilitation in a rehabilitation hospital and was able to walk with the support of another person (Hughes GBS disability grade 3/6), and a recheck of serum HBV-DNA was negative.

## 3 Discussion

HBV infection is moderately endemic in China; it is currently estimated that the prevalence of HBsAg in the general population is approximately 5% to 6%.^[1]^ HBV is transmitted through mother-to-child exposure, blood (including minor wounds on the skin and mucous membranes), and sexual contact. However, as many as 30% of hepatitis B patients fail to identify the exact mode of HBV transmission. Patients with chronic HBV infection are characterized by the presence of serum HBsAg for longer than 6 months, and serve as the main reservoir for HBV transmission.^[12]^ The patient in this case report denied a history of high-risk sexual intercourse, blood transfusion, and drug injection, and his route of HBV infection was difficult to track. Chronic HBV infection can lead to hepatic inflammatory damage and resulting abnormal liver function, and the diagnosis of acute exacerbation of CHB is considered when the serum ALT level rises to more than 10 times the upper limit of normal. Indeed, the acute exacerbation of CHB is not uncommon and its predisposing factors include the spontaneous reactivation of CHB in the natural history, the cessation of antiviral drugs such as NAs, the use of immunosuppressants or chemotherapy, HBV genotypic variation, and superinfection with other viruses such as Hepatitis A, C, and D, and human immunodeficiency virus.^[13,14]^ It is thought to be the result of a human leukocyte antigen-I restricted, cytotoxic T lymphocyte-mediated anti-HBV immune response, and its downstream mechanisms.^[14]^ The clinical spectrum of the acute exacerbation of CHB ranges from completely asymptomatic to symptomatic, to manifestations resembling acute hepatitis, which in severe cases can lead to cirrhosis and liver failure. In addition, extrahepatic manifestations including nervous system involvement may also occur. Although this patient had a history of schizophrenia, there are no documented case reports of schizophrenia directly causing GBS or evidence of a correlation between the 2 to date. Additionally, his serological indicators showed no evidence of preceding infection with other viruses or bacteria that would induce the onset of GBS. Therefore, we believe that he may have experienced a rebound of the immune system due to self-discontinuation of ETV antiviral therapy, which led to the acute exacerbation of CHB, thus causing symptoms similar to acute hepatitis B combined with GBS.

Approximately 1% of GBS episodes have been observed in association with acute hepatitis (hepatitis A, B, C, D, and E),^[15,16]^ and occasionally with CHB, such as in this case. Unfortunately, the exact mechanism by which chronic HBV infection induces peripheral nerve damage in GBS has not yet been elucidated. A neuropathological study on HBV infection-associated GBS showed positive HBsAg-immunofluorescence labeling around the endoneural small blood vessels and in the endoneurium in affected individuals, as well as significantly higher levels of HBsAg-immune complexes in both serum and CSF.^[6]^ The level of immune complexes increased with the appearance of neurological symptoms and decreased with the improvement of neurological symptoms.^[6]^ Another study revealed that immune complexes can deposit in the endoneurium through the blood–nerve barrier and injure nerve fibers,^[17]^ suggesting that they are important pathogenic agents of GBS associated with HBV infection.^[18]^ In addition, HBV may present with some similar components to peripheral nerves, and circulating immune complexes can induce an imbalance of T-cell subpopulations and decrease T-cell suppressor activity in the peripheral blood of GBS patients.^[19–21]^ HBV infection may trigger the production of autoantibodies and the activation of monocytes through molecular mimicry,^[21,22]^ which results in immune damage to myelin and axons.

A literature search using the PubMed database with the keywords “chronic hepatitis B” and “Guillain–Barré syndrome” retrieved only 4 case reports of CHB-related GBS published in English. To date, there is no English literature on CHB-related GBS cases in Chinese mainland. With the addition of our patient, there were a total of 7 cases. Table 2 shows the summarized clinical features of CHB-related GBS patients from the literature. The majority of patients were male (6 males out of 7 cases, 85.7%), with a mean age of GBS onset of 45 years (range 29–65), residing in Eastern Asia, Southern Asia, and Southeastern Europe, suggesting these as high-incidence areas. These patients showed acute hepatitis-like symptoms such as jaundice, nausea, fatigue, fever, anorexia, abdominal pain, and hepatomegaly. By the time of GBS onset, the disease course of CHB in the mentioned cases was 2 to 12 years. Subsequently, the patients showed clinical manifestations of peripheral nervous system involvement of varying severity, and related symptoms of GBS including motor weakness, cranial nerve palsy, and sensory disturbances. Six of the 7 cases (85.7%) showed a monophasic course, and only 1 patient (14.3%) presented with a recurrent GBS onset during the acute exacerbation of CHB, or a polyphasic course, suggesting that a small number of CHB-related GBS patients exhibited relapsing properties. Moreover, respiratory muscle paralysis was observed in 2 out of 7 cases (28.6%), suggesting that the condition was prone to aggravation in a small number of CHB-related GBS patients. Among these 7 cases, our patient was the only documented presentation of limb muscle atrophy combined with respiratory failure, showing that he was a rare and critical case of CHB-related GBS.

**Table 2:** Clinical characteristics of CHB-related GBS.

					Virological markers				
Reference	Country or region	Age/sex	Duration of GBS onset from CHB	Symptoms	HBsAg	HBeAg	HBeAb	HBcAb	HBV-DNA
Tsukada et al (1987)^[6]^	Japan	34/M	12 yr	Mo/Se/CP	+	−	+	+	NM
	Japan	48/F	NM	Mo/Se	+	NM	NM	NM	NM
Han et al (1999)^[7]^	Taiwan	46/M	NM	Mo/Se	+	+	+	IgM (−)	NM
	Taiwan	29/M	3 yr	Se	+	−	+	NM	+
Chroni et al (2003)^[8]^	Greece	65/M	Ep 1: NM	Mo/Se/CP/RF	+	−	+	IgM (−), IgG (+)	2235^∗^
			Ep 2: 4 yr	Mo/Se	NM	NM	NM	IgM (+)	4^∗^
Sonavane et al (2018)^[4]^	India	59/M	NM	Mo/CP	+	−	+	IgM (+)	100,890^†^
Our case 2021	Chinese mainland	34/M	2 yr	Mo/Se/MA/RF	+	−	+	IgM 1:502	110,000^†^

			CSF				
ALT/AST (IU/L)	Total bilirubin (mg/dL)	Ganglioside antibody	Protein level (mg/dL)	WBC count (×10^6^/L)	NCS	Treatment	Prognosis
65/NM	NM	NM	225	NM	AIDP	Steroid	Complete recovery
66/52	NM	NM	108	NM	AIDP	Steroid	Complete recovery
1366/750	NM	NM	92	0	AIDP	IVIG/PP	Marked recovery
761/383	1.7	NM	60	1	ASN	IFN-α	Complete recovery
154/125	0.9	NM	125	0	AIDP	MV/prednisolone/PP/IVIG	Complete recovery
308/156	1.2	NM	70	0	AIDP	Lamivudine/IVIG	Complete recovery
1345/910	14.6	NM	247	10	AIDP	Entecavir/IVIG	Complete recovery
231/204	5.0	GD1a, GQ1b, GM1 and GM2 (-)	72	2	AMSAN&AIDP	TDF/IVIG/MV/hepatoprotection/acupuncture and rehabilitation	Partial recovery

CHB is a dynamic pathological process of long-term interaction among HBV, host hepatocytes, and immune cells. HBV serological indicators and HBV-DNA can reflect the current viral infection status and replication level, and ALT and AST levels can reflect the degree of liver cell damage to a certain extent. Literature review of the 7 reported cases showed that the serum ALT and AST levels of patients were elevated. Among them, 2 patients with mild liver dysfunction were diagnosed with chronic active hepatitis (virological markers: HBsAg positive, HBeAg negative, HBeAb positive, and HBcAb positive) and chronic persistent hepatitis (serological HBsAg positive) by liver biopsy, suggesting that the latter 2 may be the pathological basic phenotypes responsible for the onset of GBS. It has been documented that patients with the acute exacerbation of CHB exhibit a dramatically rising level of HBV-DNA before the elevation of serum ALT,^[23,24]^ and the serum HBsAg level also rises in parallel with the elevation of serum HBV-DNA.^[14]^ One of the 7 patients with an abrupt rise in liver enzyme level was diagnosed with chronic active hepatitis associated with acute exacerbation via liver biopsy (virological markers: HBsAg positive, HBeAg negative, HBeAb positive, and HBV-DNA elevated). The remaining 4 patients with a sharp elevation of liver enzyme level but no liver biopsy (serological HBsAg positive, HBeAg positive or negative, HBeAb positive, HBcAb IgG positive and IgM negative or positive, and HBV-DNA elevated) were reported to have an acute exacerbation of CHB, suggesting that the acute exacerbation of CHB may be a major predisposing factor for GBS onset. In our case, a sharp increase in serum ALT/AST levels was almost accompanied by an MRC-sumscore decline, and vice versa, also demonstrating the close association between acute exacerbation of CHB and GBS onset. In addition, elevated serum total bilirubin was noted in 3 of the 7 patients reviewed, and normal total bilirubin was noted in 2, indicating that jaundice may be a common but not necessarily presenting symptom of CHB combined with GBS.

GBS is a neuropathologically diverse disorder that includes several unique subtypes such as AIDP, acute motor axon neuropathy, AMSAN, Miller–Fisher syndrome, acute panautonomic neuropathy, and acute sensory neuropathy. CSF examination and NCS play an important role in improving diagnostic accuracy and for judging the therapeutic efficacy and prognosis of GBS. Analysis of CSF generally shows an albuminocytologic dissociation phenomenon (an elevated protein level and a normal white blood cell count), especially in the third week of the disease course. The 7 CHB-related GBS patients all showed this characteristic in their CSF. NCS revealed that 5 of these 7 cases (71.4%) belonged to AIDP, 1 to acute sensory neuropathy, and our reported case to AMSAN with concomitant AIDP, suggesting that AIDP is the most frequent neuro-electrophysiological subtype of CHB-related GBS. It has been documented that less than 10% of GBS cases belong to the AMSAN subtype, of which patients are usually in critical condition and prone to respiratory muscle weakness, muscle atrophy in the limbs, and slow recovery with poor prognosis.^[25]^ These features were fully demonstrated in our patient, who was the first reported rare case with both the AMSAN and AIDP electrophysiological subtypes in CHB-related GBS. In addition, serum antiganglioside antibodies GM1 and GDla have been documented to be positive in a subset of AMSAN patients,^[26]^ which may deepen the understanding of the pathogenesis of immune-mediated GBS. However, not all patients were positive for these antibodies, as was seen in our case. In future studies, it is necessary to increase the number of CHB-related GBS patients tested for antiganglioside antibodies to determine the correlation between the two.

IVIG and plasmapheresis (PP) are generally considered first-line effective treatments for GBS.^[27,28]^ IVIG can neutralize circulating antibodies, block Fc receptors, and exert immunomodulation on B and T cells,^[29]^ and PP can eliminate circulating antibodies and complements and improve T-cell suppressor function.^[30]^ Both oral and intravenous corticosteroids are considered potentially ineffective in the treatment of GBS.^[31]^ Although combined treatment with IVIG or PP may accelerate neurological recovery in the short-term, corticosteroids are not beneficial to the long-term prognosis of patients^[32]^ and not recommended as a standard first-line therapy for GBS. Among the 4 reported cases with available details of IVIG and (or) PP treatment, 2 recovered completely, 1 improved markedly, and 1 improved partially. Surprisingly, 2 patients treated with corticosteroids but not IVIG or PP recovered fully, and 1 patient completely recovered with interferon-α (IFN-α) alone (no IVIG, PP, or corticosteroid treatments). The effect of these nonstandard individual therapeutic measures on CHB-related GBS should be evaluated in further clinical epidemiological investigations. In our case, the treatment method differed slightly in that the patient was given 2 courses of IVIG to effectively prevent the progression of the disease. Some believe that severe GBS patients experiencing poor outcomes after the first course of IVIG may benefit from a second course of treatment,^[33–35]^ which indeed occurred in our patient.

The treatment of CHB is another important concern in CHB-related GBS. Early intervention with antiviral drugs inhibits the replication of HBV and reduces the formation of HBsAg immune complexes, thereby alleviating the latter's damage to nerves and possibly improving outcomes. CHB patients with positive serum HBV-DNA require active antiviral therapy if other causes of persistently elevated ALT level have been excluded. NAs such as ETV, TDF, and lamivudine are common anti-HBV drugs. Peg-IFN-α and IFN-α are also recommended for the treatment of CHB. Among the 4 cases with available details of antiviral treatment, 3 (75.0%) were treated with NAs combined with IVIG or PP, and only 1 (25.0%) with IFN-α alone, suggesting that NAs combined with IVIG or PP may be the core therapeutic strategy for CHB-related GBS. It is noteworthy that during the treatment of CHB, NAs therapy should not be discontinued arbitrarily, because serum HBV-DNA may rise in response to the resumption of HBV replication after cessation of therapy, thereby potentially precipitating the acute exacerbation of CHB,^[36–39]^ as evidenced by our patient who self-discontinued ETV 8 months prior to hospitalization. In addition, hepatoprotective therapy is beneficial to alleviating the inflammatory response in hepatocytes and promoting the recovery of liver function, and is an important supportive treatment measure for CHB. In our case, the oral NA TDF combined with hepatoprotective therapy successfully halted the continued deterioration of CHB.

Other comprehensive therapeutic measures for patients with CHB-related GBS should include airway management, psychosocial support, physiotherapy, and rehabilitation. Two of the 7 cases reviewed, including ours, received emergency mechanical ventilation to avoid the risk of death from respiratory failure. Due to chronic schizophrenia, our patient also received antipsychotic treatment and psychological support provided by psychiatrists to avoid possible negative effects of the psychomental disease on the treatment of his somatic disease (GBS). Our patient also received acupuncture and limb exercise therapy, which was helpful for promoting the early recovery of nerve function to a certain extent.

## 4 Conclusions

CHB-related GBS is a rare disease. This case report describes for the first time a severe case of CHB-related GBS with the neuro-electrophysiological characteristics of AMSAN with concomitant AIDP. GBS should be included in the list of rare extrahepatic manifestations of CHB, and the acute exacerbation of CHB may be a potential predisposing factor for the onset of GBS. Early diagnosis and prompt treatment with the goal of striving for favorable and long-term outcomes in clinical practice are difficult and challenging. During the acute exacerbation of CHB, physicians should focus on the diagnosis of CHB-related GBS in patients with symptoms of limb weakness and numbness when other common causes are excluded. Early administration of IVIG or PP in combination with antiviral agents and supplemented with hepatoprotective treatment, acupuncture, and rehabilitation may allow rapid and better recovery in patients with CHB-related GBS. The theragnostic strategy in our case may provide a reference for clinicians.

## Author contributions

**Conceptualization:** Jiajun Wei.

**Data curation:** Jiajun Wei.

**Formal analysis:** Jiajun Wei.

**Investigation:** Shenhan Duan, Jiajun Wei

**Project administration:** Jiajun Wei.

**Supervision:** Jiajun Wei

**Resources:** Jiajun Wei, Shenhan Duan

**Writing – original draft:** Jiajun Wei.

**Writing – review & editing:** Shenhan Duan, Jiajun Wei.
